# Newly Discovered Archival Data Show Coincidence of a Peak of Sexually Transmitted Diseases with the Early Epicenter of Pandemic HIV-1

**DOI:** 10.3390/v13091701

**Published:** 2021-08-27

**Authors:** João Dinis Sousa, Philip J. Havik, Viktor Müller, Anne-Mieke Vandamme

**Affiliations:** 1 Clinical and Epidemiological Virology, Rega Institute for Medical Research, Department of Microbiology, Immunology and Transplantation, KU Leuven, B-3000 Leuven, Belgium; annemie.vandamme@kuleuven.be; 2 Global Health and Tropical Medicine, Instituto de Higiene e Medicina Tropical, Universidade Nova de Lisboa, 1349-008 Lisbon, Portugal; philip.havik@ihmt.unl.pt; 3 Institute of Biology, Eötvös Loránd University, 1117 Budapest, Hungary; mueller.viktor@ttk.elte.hu; 4 Institute for the Future, KU Leuven, B-3000 Leuven, Belgium

**Keywords:** HIV, HIV-1, origin of HIV, zoonosis, pandemics, enhanced heterosexual transmission hypothesis, sexually transmitted diseases, central Africa

## Abstract

To which extent STDs facilitated HIV-1 adaptation to humans, sparking the pandemic, is still unknown. We searched colonial medical records from 1906–1958 for Leopoldville, Belgian Congo, which was the initial epicenter of pandemic HIV-1, compiling counts of treated STD cases in both Africans and Europeans. Almost all Europeans were being treated, while for Africans, generalized treatment started only in 1929. Treated STD counts in Europeans thus reflect STD infection rates more accurately compared to counts in Africans. In Africans, the highest recorded STD treatment incidence was in 1929–1935, declining to low levels in the 1950s. In Europeans, the recorded treatment incidences were highest during the period 1910–1920, far exceeding those in Africans. Europeans were overwhelmingly male and had frequent sexual contact with African females. Consequently, high STD incidence among Europeans must have coincided with high prevalence and incidence in the city’s African population. The data strongly suggest the worst STD period was 1910–1920 for both Africans and Europeans, which coincides with the estimated origin of pandemic HIV-1. Given the strong effect of STD coinfections on HIV transmission, these new data support our hypothesis of a causal effect of STDs on the epidemic emergence of HIV-1.

## 1. Introduction

The Human Immunodeficiency Virus type 1 (HIV-1) has a pandemic strain (group M) that descends from Simian Immunodeficiency Virus (SIV) infecting common chimpanzees (*Pan troglodytes troglodytes*; SIVcpz). Although there are other epidemic HIV strains [1,2,3,4,5], in this article, we focus on HIV-1 group M only. Phylogenetic analyses of SIVcpz/HIV-1 suggest that the initial chimpanzee-to-human transmission occurred in southeast Cameroon, likely through bushmeat handling activities [1,4,6,7,8]. The time of the most recent common ancestor (tMRCA) of HIV-1 group M has been dated to the early 20th century. Early pioneering studies dating the tMRCA suggested a timing around 1930, at 1931 (confidence interval: 1915–1941) [9], 1920 (1903–1939), and 1937 (1925–1949) [10]. The latest analyses, which incorporate the findings of several fossil viruses recovered from stored samples, suggest a timing closer to the start of the 20th century, dating the tMRCA at 1908 (1884–1924) [11], 1920 (1909–1930) [12], 1906 (1892–1921) [13], 1920 (1915–1925) [14]. Phylogeographic analyses with hundreds of HIV-1 group M sequences collected from many different Central African cities revealed that the epidemic very likely started in Kinshasa [12,13]. Kinshasa (called Leopoldville in colonial times) is the capital of the Democratic Republic of Congo (formerly the Belgian Congo). The implication is that sometime in the late 19th or early 20th century the ancestral SIVcpz virus crossed to humans in Southeast Cameroon and then one or a few infected humans brought the virus to colonial Leopoldville, where conditions allowed it to spread, diversify, and adapt to the human host species [2,4,6,7,8].

Humans have long been exposed to zoonotic SIV infections in Africa. SIVs appear to be at least 77,000 (33,000–133,000) years old [15]. Two studies involving thousands of Central African people exposed to bushmeat found 8–17% SIV seroreactivity, its frequency correlated with exposure. No viruses could be isolated or sequenced [16,17]. Thus, human exposure to SIV infections is frequent, but mostly unproductive and the viruses are not transmitted between humans, indicating lack of adaptation to the human organism. In contrast, there is solid evidence that epidemic HIV strains have adapted to the human host and that HIV-1 group M is the best adapted one. HIV-1 groups M, N, and O, and HIV-2 group A have all evolved an arginine at the Gag30 position (changing from methionine in their SIV ancestors), and this convergent change constitutes an adaptation to the human host [4,18]. HIV-1 groups M and O, and HIV-2 have all evolved the capacity to antagonize human tetherin, and this antagonism is strongest in HIV-1 group M [4,19,20]. Epidemic HIV groups also evolved the capacity to downregulate human CD4, HLA-A, HLA-B, and HLA-C [4,21]. All this evidence together suggests that the epidemic success of the few emergent HIV strains was only possible through adaptation to the human host.

The long-lasting human exposure to SIV and absence of HIV emergence before late 19th–early 20th century suggests that SIV adaptation to humans is unusual and difficult, and the emergence of several epidemic HIV strains in a short time window in the early 20th century was probably driven by novel factors appearing in Central and West Africa at that time. This assumption was made by most researchers investigating the origins of HIV [6,7,8,11,22,23,24,25,26,27,28,29,30,31]. The changes brought about by effective colonial occupation (starting around 1885) that have been tentatively implicated in HIV adaptation and emergence were urbanization and associated social changes [11,31], medical injections, smallpox vaccinations, transfusions [23,24,25,26,27,28], and the spread of sexually transmitted diseases (STD), including genital ulcer diseases (GUD) [6,7,8]. Serial transmission is an effective way to improve viral adaptation to a new host species [4,24,32]. In the case of HIV, it might have been enabled by the presence of co-factors known to enhance HIV transmission, such as injections and STD, because unsterile injections and transfusions [33,34], and STD, particularly GUD [35,36,37,38], strongly increase current HIV transmission probability in relation to baseline sexual transmissibility. We have previously shown that, like injections and transfusions, epidemic GUD was also absent from interior Central Africa, in late 19th–early 20th century [6,8,39,40]. Epidemic GUD thus fits the above-cited criterion of novelty. We recently contributed a follow-up on the state of this debate [8].

## 2. Added Value of the Present Study over Our Previous Archival Research

In our previous work [6,40], we built temporal statistics of incidence rates for STD and GUD treated cases for the period 1919–1958 in colonial Leopoldville, based on nosological tables and demographic data extracted from the Belgian archives covering the colonial period (1885–1960). Briefly, we found that the treatment incidence rates of the ulcerative stages of GUDs (primary and secondary syphilis, caused by the bacterium *Treponema pallidum pallidum*; chancroid, caused by the bacterium *Haemophylus ducreyi*; and lymphogranuloma venereum or LGV, caused by the L1–L3 serotypes of the bacterium *Chlamydia trachomatis* [41]) were two orders of magnitude higher in the period 1919–1935 than in the late 1950s. The strong decline seen in STDs and GUDs in Leopoldville is entirely explained by the increase in availability, coverage, and efficacy of treatments, first with the establishment of an STD-specialized Croix Rouge clinic, which provided unprecedented levels of treatment and screened the majority of the female population in the early 1930s [42], followed by the arrival of sulfonamides in 1937, and by the adoption of antibiotics in the late 1940s [42,43,44]. Therefore, GUD peaked in Leopoldville in the early 20th century, when treatments were less effective, showing a fair coincidence with the tMRCA datings of HIV-1 group M that had been published up to the time of our publication (central estimates between 1908 and 1937) [9,10,11].

In a modeling paper [6], we proposed that novel epidemic GUD may have been the factor triggering the adaptation and emergence of several epidemic HIV groups all in the early 20th century, additionally explaining lack of HIV emergence during tens of thousands of years of human exposure to SIV through bushmeat handling [6]. However, that study was based on STD and GUD data that were incomplete. We based our modeling on counts in Africans only, and for them we were not able to find valid data preceding 1919.

The present study extends the survey to Europeans, and we also collected some limited additional data for Africans. As before, for Africans we were not able to find any valid data for any year before 1919, while the data for Europeans did allow us to obtain reliable pre-1919 data. This is timely, because in the most recent studies of the tMRCA of HIV-1 group M a high probability density falls before 1919 [11,12,13]. Europeans were almost always treated for STDs, implying that treatment records present a reliable data source on the overall STD incidence in this group.

## 3. Materials and Methods

### 3.1. Overview of the Reports Studied

We searched colonial archives and articles for quantitative data on treated STD cases in Leopoldville. The archives searched were the African Archives of the Belgian Ministry of Foreign Affairs, which absorbed the Archives of the old Ministry of Colonies (Afrika-Archief, Federale Overheidsdienst—Buitenlandse Zaken, Buitenlandse Handel en Ontwikkelingssamenwerking, FO-BZBHO). This research was performed in 2016–2018. We concentrated on colonial health reports, general reports containing health related sections, and similar documents, about the Belgian Congo. We focused on the city of Leopoldville and tried to obtain STD incidence data for the entire colonial period (1885–1960). We complemented this archival research with a detailed review of colonial medical articles.

All reports reviewed are annual and written in French. Most were titled ‘*Rapport du Service Médical*’ or ‘*Rapport du Service d’Hygiène Publique*’. Some of them are general reports containing extensive sections about health (e.g., ‘*Rapport Annuel sur l’Administration de la Colonie du Congo Belge*’). Most reports from 1919 onwards, and some from before that, include nosological tables with counts of disease cases treated in the city or region concerned, in a particular year. The reports refer to the colony level, province level, district level, or city level.

Up to 1922, many reports list city-level data for Leopoldville itself (*Cité de Léopoldville*, *Station de Léopoldville*), even when they also describe district-level data for the Moyen Congo district (to which Leopoldville belonged). From 1923 onward, a special urban district comprising exclusively the city of Leopoldville was defined (*District Urbain de Léopoldville,* DUL), and there is a complete series of health reports for the DUL [43]. Their data is at DUL level, equivalent to city level. Other reports concern the entire province of Congo-Kasai, in which Leopoldville/DUL was included until 1932 [44]; from 1933 onwards, the provinces were re-configured, and Leopoldville/DUL was thereafter included in the Leopoldville Province [45]. Many of these province-level reports break down the disease case counts by the districts comprising the province, including the DUL, which allowed for the extraction of city-level data. Some of the colony-level reports also have counts broken down by provinces and districts, and we extracted city-level data from them [46,47,48,49]. We obtained demographic data for the city, from the health reports above described, and from additional sources [50,51,52,53,54,55,56,57,58,59]. Although we consulted most reports at the Afrika-Archief, for some we obtained printed copies from libraries. The full list of archival and other sources we consulted to retrieve, respectively, disease counts and demographic data, is presented in Supplementary Tables S1 and S2.

### 3.2. The STD Data Collected

We were interested in disease counts of both hospitalized patients and patients treated in dispensaries or clinics, since most STD cases do not require hospitalization. The reports usually, but not always, contained four extensive nosological tables with the counts of disease cases treated in public sector health facilities, combining two categories, one for hospitalized or interned cases only (‘*hospitalisés*’) and the other for all cases whether hospitalized or not (‘*hospitalisés ou non*’), and the two main social strata, Africans (‘*Noirs*’) and Europeans (‘*Blancs*’), reflecting the strict racial segregation in healthcare typical of colonial systems. We used only all-cases (‘*hospitalisés ou non*’) nosological tables to compile our database. If only the table of hospitalized cases was available, we regarded this as missing data for that year. Importantly, these tables contained counts of *disease cases* that were treated, regardless of the number of *consultations* involved. A certain disease case treated in a year could involve several visits or consultations for examination or treatment. The reports are very clear in distinguishing disease cases from consultations, and we focused on the former. In our 2010 study [6], we compiled data only on Africans. In the present study we mainly compiled data for Europeans, but still added some data for Africans that were not included in our previous study. For Europeans, we used public sector nosological tables, because they were almost always treated in public sector facilities, and only these tables were available for them. For Africans, we added the cases treated by private companies (‘*Higiène Industrielle*’ tables [43]) and the Red Cross clinic (‘*Croix Rouge du Congo*’ tables [42]), as we did in our previous study (see Supplementary Text S1).

We compiled annual counts of treated cases of the following STD conditions (those reported consistently): syphilis (all stages), syphilis (primary and secondary stages), chancroid, the venereal bubo syndrome (bubon vénérien; typical of secondary LGV, mostly seen in males), and urethral/cervical discharge syndrome (described as ‘*blennorrhagie*’). The latter is most often caused by the bacteria *Neisseria gonorrhoeae* or *Chlamydia trachomatis* [60]. In Leopoldville, the causative role of *Chlamydia trachomatis* and other pathogens was not known for most of the period under study and attempts to detect specific pathogens by cell culture were not made. Thus, the reports only mention ‘*blennorrhagie*’ to encompass all cases of discharge syndrome, and we cannot break down the cases by causative pathogen, and so we collected and present in this study the reported number of cases of ‘*blennorrhagie*’.

### 3.3. Calculation of Incidence of Treated Cases

For some years, we could not obtain the population census for the city, but we obtained it for the Moyen Congo district, and we estimated the census for the city based on the relative proportion of the city within the district, linearly interpolated between years for which Moyen Congo data existed. For most years, the archival sources provided the breakdown of the population into men, women and children. When this information was lacking, we estimated their proportions (see Supplementary Text S1).

For each STD, for each year for which we had both demographic and STD data, we calculated the annual incidence of treated cases as:

incidence = disease count/adult population

We calculated the incidence of primo-secondary syphilis separately from syphilis in all stages because we have good data on the proportion of syphilis cases that were treated in primary and secondary stages, including its variation over time, obtained in our previous study [6].

For the years 1910, 1911, and 1914, we made estimates for syphilis based on quantitative statements written in the reports. We discuss the criteria in the Supplementary Text S1.

In general, treated chancroid has sex ratios between 3:1 and 25:1 [61,62]. For the venereal bubo syndrome of LGV, the sex ratios vary between 4:1 and 22:1 [63]. Since the STD data in our nosological tables is not broken down by gender, we assumed a sex ratio of 9:1, intermediate between the above cited statistics. The choice of 9:1 is ad-hoc, but has little effect on the trend of the incidence of treated cases. Based on this, for chancroid and LGV venereal bubo we calculated incidence as:

incidence = disease count × 0.9/adult male population

For the other STDs we divided the number of cases by the total adult population. We did not perform direct interpolation on incidence rates.

### 3.4. Proportion of Syphilis Cases Treated in Primary or Secondary Stage

The reports contain information on the partition of treated syphilis cases by stage of syphilis for extended periods. We were interested in the proportion of cases detected in either the primary or the secondary stage because only in these stages syphilis causes genital ulcers. Since this information was lacking for many years, data for the missing years were imputed using a smooth function constructed through local polynomial regression fitting over the available data points, as implemented in the loess() function of the R language [64]. We give details on this in Supplementary Text S1, Section 6, and provide the R script as Supplementary Script S1.

### 3.5. Estimate of Male Circumcision Rate

Previously, we had estimated the male circumcision rate in Leopoldville’s Africans [6]. Now, we recalculate the estimated rate for the whole male population, including Europeans, based on our previous estimate for Africans, taking into account the proportion of males that were European, and assuming a 2% circumcision rate among Europeans (consistent with existing data [65,66]). We give details about this calculation in Supplementary Text S1, Section 7.

## 4. Results

### 4.1. STD Incidence in Europeans Peaked in the Period 1910–1924

The level of STDs in Europeans was probably strongly linked to their level in Africans, since Europeans were overwhelmingly male and had many contacts with African CSWs and other African women [67,68]. In contrast to Africans, Europeans had much better access to health care, the anti-syphilis drug *NeoSalvarsan* was generally available to them, and so counts of treated and recorded cases are probably closer to the real number of infections, even for years previous to 1929. In addition, pre-1919 nosological tables on Europeans are available in the archives, while, for Africans, such data is lacking (Supplementary Table S1).

**Figure 1:**
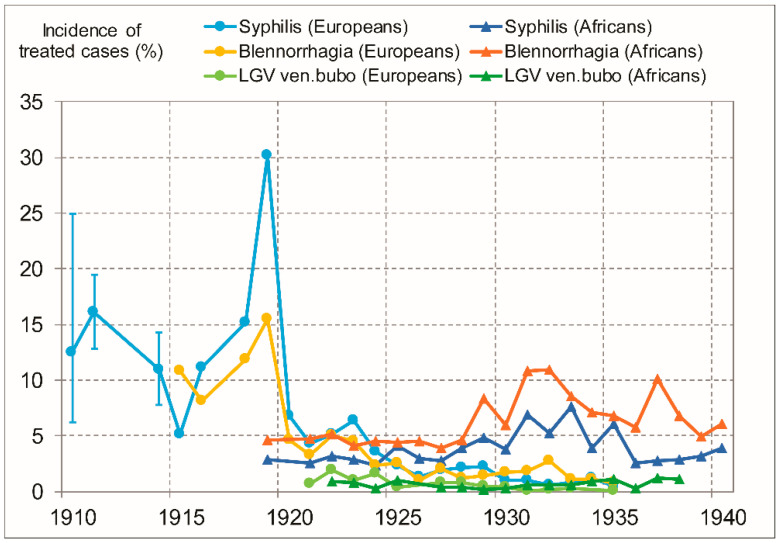
Incidence of treated cases (in %) of several STD conditions in Leopoldville’s European and African adults (only males for chancroid and LGV venereal bubo). For details, see main text. The data supporting this figure are presented in Supplementary Dataset S1. The error bars for the years 1910, 1911, and 1914 for syphilis represent our lower and upper estimates, explained in Supplementary Text S1. For years without error bars, the data point represents the single reported value extracted from the archives.

We found useful nosological tables (including hospitalized or not) for the entire period 1919–1935, and for 1915, 1916, and 1918. For the period 1935–1958 only province-level nosological tables were available for Europeans. Other quantitative descriptions allowed us to make estimates for 1910, 1911, and 1914 (explained in Supplementary Text S1). LGV data is available only from 1921 onwards and chancroid data only from 1930 onwards. We present counts of treated STDs, demographic data, and incidence of treated cases in Supplementary Dataset S1. For the period 1910–1940, we display the incidence of treated cases for the various STDs in Europeans and in Africans in Figure 1.

As the figure shows, the incidence of treated cases of syphilis and discharge in Europeans peaked before 1920, remained high until 1923, and dropped significantly after that. LGV incidence declined after 1924. Clearly, the rates in Europeans, in the period 1910–1919, were higher than corresponding rates at any other time, for Europeans or Africans. LGV peaked in 1921–1924, the earliest years for which data exist. By 1935, syphilis, discharge, and LGV had declined by about one order of magnitude.

**Figure 2:**
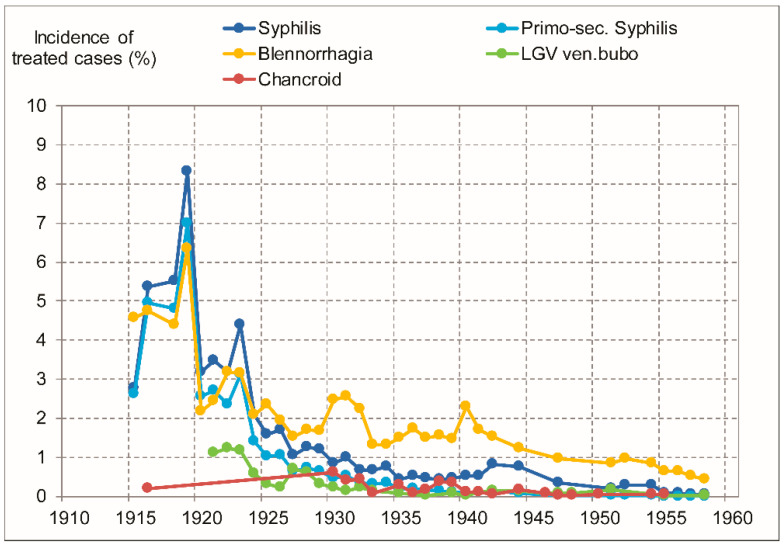
Incidence of treated cases (in %) of several STD conditions in European adults of the province that included Leopoldville (only males for chancroid and LGV venereal bubo). Chancroid data was published regularly only after 1930, and, before that, only once, in 1916. For details, see main text. The data supporting this figure are presented in Supplementary Dataset S1.

For the province as a whole, we could obtain data for Europeans spanning the period 1915–1958 and we observed that incidence rates, by the late 1950s, had dropped one (chancroid and discharge) or two (syphilis and LGV) orders of magnitude (Figure 2). The proportion of syphilis detected in primary or secondary stages declined strongly, as predicted by syphilis epidemiological models [69]. These patterns are similar to those found for Leopoldville’s African population. Briefly, and as we published elsewhere [6,40], the incidence of treated cases of ulcerative stages of GUD in Africans declined by two orders of magnitude between 1919–1935 and the late 1950s, as STD treatment campaigns were implemented, and sulfonamides and antibiotics were adopted.

The incidence of treated cases of STDs in Europeans in 1910–1919 was clearly higher than the corresponding incidences in Africans in 1929–1935 (Figure 1). At the time, mass screening was available and treatment coverage was high and probably comparable to that for Europeans. Since Europeans were mostly acquiring their STDs from African CSWs and other women, the levels of STD infections in Africans in 1910–1919 were also probably higher than in 1929–1935 or 1919–1928.

Male circumcision is an important factor that reduces female to male transmission of HIV, syphilis, chancroid, and other GUDs [70,71]. Previously, we had estimated the male circumcision rate in Leopoldville’s Africans, in 1919, as being in the range 73.5–82.5% [6]. Now, we recalculate the estimated rate for the whole male population including Europeans. Our new estimate is 70.4–79.0% (calculation explained in Supplementary Text S1, Section 7).

### 4.2. Lack of Adequate Treatment Explains Lower Recorded Incidence of Treated Cases in Africans, in 1919–1928

For Africans, Figure 1 shows a surge in treated STD cases from 1929 onwards, but this reflects the opening of the Croix Rouge clinic and associated mass screening and treatment [6,40], rather than real epidemic growth. Before 1929, no comparable clinic existed, and we gathered evidence that STD treatments in Africans were less common than in the later period of 1929–1935 and also of lower quality. Gonorrhea and chancroid treatments were still very ineffective, making patients less keen to seek treatment. The anti-syphilis arsenical drug *Neoarsphenamine* (*NeoSalvarsan*) already existed, but was used sparingly in Africans. Hence, in 1922, for 492 syphilis cases in Africans treated in Leopoldville, only 160 injections were performed [44], i.e., an average of only 0.3 per patient (in contrast with >10 after 1929). In 1921, a colonial physician reported that his supplies of *NeoSalvarsan* to treat syphilis in Boma (at that time the colonial capital) were “only enough for Europeans” [72].

In the early 1920s, the lower availability of anti-syphilis drugs and the inadequacy of existing treatments against the other STDs was probably reducing Africans’ motivation to seek health care. It should be kept in mind that even nowadays, when effective antibiotic treatments are available, only 40–60% of people suffering from discharge or GUD in Africa seek health care [73]. In 1920s Leopoldville, Africans tended to be relatively careless (“insouciant”) in relation to STD symptoms [44]. In contrast, a later observation from 1933 bears testimony of Africans being increasingly aware of the relevance of the syphilitic primary chancre and increasingly seeking treatment for it [74].

Therefore, we hypothesize that a high proportion, or perhaps most STD infections in the period 1919–1928 were not detected by the health services, thus not being reported, and not reflected in our African data series shown in Figure 1. Corroborating this hypothesis, several colonial reports suggest that the STD situation in Africans was worse in the 1910s and 1920s than in the 1930s [75,76,77,78].

### 4.3. Other Evidence Supporting a Peak in STDs before 1925

Other lines of evidence suggest that the highest STD intensity happened in the early 1920s or before, in both Europeans and Africans. Several articles and reports suggest this, although not presenting nosological tables [44,75,76,77,78,79,80,81,82,83,84]. Similarly, high STD intensity was seen in European colonial and military personnel in other accounts of the same period [85,86,87]. We present these statements and their interpretation in Supplementary Text S1, Section 9, and Supplementary Table S5.

## 5. Discussion

The principal results of our study are that the incidence of treated cases of syphilis and discharge syndrome among Europeans in Leopoldville peaked in the period 1910–1919, and LGV peaked before 1925 (Figure 1). Treated syphilis annual incidence rates in Europeans were in the range 5–30% (Figure 1). The staggering jump to 30.2% incidence in 1919 may be related to more screening and treatment efforts (Wassermann tests started to be applied regularly at about that time [88]). Even without this peak value, the incidence rates were in the range 5–15%, high even by early 20th century standards, but not unrealistic: a review paper of STDs in the military shows similar figures in some colonial troops [86]. These rates are clearly higher than the highest rates reported in Africans, throughout the period 1919–1935 (Figure 1). For the period between 1919 and 1928, the recorded rates of treated cases in Africans were likely not representative of real STD incidence, because Africans, at the time had little access to treatments.

The incidence rates for Africans before 1919 cannot be established owing to a lack of relevant nosological tables in the Archives, but the peak rates among Europeans in 1910–1919, together with the fact that European men had sexual contacts primarily with African women suggest that this period was the most acute in terms of incidence of STD infection, also for Africans, in the entire colonial period. Supplementary Table S5 provides further evidence.

High infection rates imply high prevalence of the initial stages of STDs; these include genital ulcers (typical of primary and secondary syphilis, chancroid, and LGV), and discharge syndrome (typical of gonorrhea and chlamydia) that increase the probability of HIV transmission.

The ancestor virus of HIV-1 group M likely arrived in Leopoldville at that time from southeast Cameroon, where it first crossed from chimpanzees to humans [2,4,6,7,8]; regular lines of steamers linked the two locations from ~1900 [7]. The high frequency of genital ulcers and discharge conditions probably gave a decisive boost to sexual serial transmission of a still ill-adapted HIV virus, promoting its adaptation and emergence as today’s recognized pandemic HIV-1 group M [6,7,8]. The co-factor effect of genital ulcers on HIV transmission rate is much higher than that of urethral discharge [37,38], but the latter had a higher prevalence in the population [76,77,78,82] and thus both may have been important.

We also showed earlier that among Leopoldville’s Africans male circumcision was less common in early 20th century, in contrast with mid-20th century, when it became nearly universal, remaining so until the present day [6]. This change increased HIV transmission probability from females to males, and thus increased the odds of HIV serial transmission and emergence. We must add that African and European men shared access to the same communities of CSWs and other sexually active African women, and thus it is justified to calculate male circumcision rates for all combined. The uncircumcised status of most Europeans may partly explain their high incidence of syphilis in 1910–1920, since circumcision reduces the female-to-male transmission probability of syphilis [71].

In our 2010 study, we showed that STD and GUD intensity in Leopoldville had peaked in the pre-1935 period [6], but we had data only for the years from 1919 onwards. With the present study, we gather evidence that STD and GUD intensity was also high and most probably higher between 1910 and 1919 (Figure 1 and Figure 2; Supplementary Table S5). It is interesting to note that the more recent phylogenetic analyses that dated HIV-1 group M tMRCA, narrowed down the confidence intervals and pointed to earlier dates in relation to previous studies. Recent studies have central estimates ranging from 1906 to 1920 [11,12,13,14], thus corresponding closely to the period of maximal GUD and STD intensity and minimal circumcision rate.

The main alternative view, that HIV adaptation was fueled by non-sterile injections [24,25,26,27] remains plausible. The high STD intensity period, which happened before 1929, more likely in the 1910s, shows a stronger coincidence with the recent tMRCA estimates of HIV-1 group M [11,12,13,14] than the peak of injections, which appears to have occurred after 1929, when the Croix Rouge clinic started to administer injection intensive regimens to many people [42]. While a better coincidence lends credence to our hypothesis that HIV-1 might have adapted to humans through STD- and GUD-fueled sexual transmission, we recognize that the injection hypothesis remains a possibility because injection intensity was high, even before 1929, for some limited categories of people, such as hospitalized trypanosomiasis patients [89,90]. If our hypothesis is correct, HIV-1 group M may have originated in the period 1910–1919 because this was the earliest period during which several conditions facilitated serial transmission of SIV were coinciding. Our Enhanced Sexual Transmission hypothesis [6,7,8] has, nevertheless, the advantage of parsimony, since it proposes that HIV adapted to the human species by the same processes that mostly fuel its ongoing transmission in Africa, which is overwhelmingly heterosexual. Even if an initial parenteral chain of serial transmission was necessary for the initial adaptation of ancestral HIV, it is probable that the resulting partially adapted strain would have needed significant further sexual transmission to adapt to human genital mucosa and turn into a pandemic-capable pathogen [8].

Finally, we note that the extremely high incidence of STDs in the colonial European population suggests the possibility that the same network of contacts might have enabled the transmission also of ancestral HIV strains to Europeans, who might then have carried the virus back to Europe. The absence of evidence of HIV in Europe before 1970 might be explained by the apparently limited heterosexual spread of the virus in most European populations even today—the ancestral strains that were not yet adapted to humans likely had even lower transmissibility, resulting in the extinction of these early introductions to Europe. It would nonetheless be an interesting avenue for future research to look for (possibly rare) medical case reports, relating to European settlers with STDs, describing symptoms compatible with AIDS. Or, alternatively, to analyze stored medical samples from the 1920s to 1950s (accounting for the long latency of the infection), primarily from Belgium that had the strongest colonial links to the epicenter of HIV-1 group M.

Our study has some limitations. First, the short-term fluctuations in treatment incidence rates do not necessarily correspond to fluctuations in incidence of infections; instead they often derive from changes in the intensity of screening and treatment efforts by doctors. However, these short-term fluctuations are unlikely to alter our main conclusion that the period 1910–1919 had a very high, and most likely the highest STD infection rates. If anything, healthcare improved over time, and STD-affected Europeans had no less access to treatment in the 1920s and 1930s than in the 1910s, and so the drop in incidence of treated cases seen in Figure 1 and Figure 2 likely reflects a drop in infection rates.

Second, in our calculation of incidence of treated cases, we divided disease counts by the adult resident population of Leopoldville, despite the fact that the city received many visitors (state agents, military personnel, traders) who were also treated in its health facilities [91,92]. Thus, the ideal denominator should include this floating population and not only the resident population, but unfortunately, we only have quantitative information about the latter. Including the floating population would cause a moderate decrease in the calculated incidence rates. Assuming that the floating/resident ratio was probably not very different in the 1910s than later, our main finding of highest STD intensity in the period 1910–1919 remains valid.

Third, although we demonstrated that incidences of treated cases of syphilis and discharge in Europeans were highest in 1910–1919, pre-1919 incidence data on Africans is still lacking. However, the very high levels of STDs in African workers revealed by the 1911–1912 surveys (Supplementary Table S5) argue that they too, contacted CSWs very frequently, and suggest that, in that period, the STD situation was dire for both Africans and Europeans.

We have found a spatial and temporal association between early HIV-1 group M spread and a peak of STD incidence. We recognize that this association does not constitute a direct proof that STDs had a causative role in HIV-1 adaptation and emergence. In fact, any factor that facilitated STD transmission in general, would have facilitated also the serial transmission (and therefore, early adaptation) of an ancestral HIV lineage. The observed spatial and temporal coincidence reinforces our hypothesis [7,8] that HIV-1 group M emerged under circumstances where and when it was particularly likely to do so by the means of heterosexual transmission, and our earlier modelling work [6] suggests that the facilitating effect of GUD co-infections might have played an important role in the process.

## 6. Conclusions

In this article, we were able to demonstrate for the first time that the period of maximal GUD and STD intensity in Leopoldville occurred around 1910–1919, with incidence rates that were impressive even by early 20th century standards. Such timing coincides with the most recent estimates of the origin of the HIV-1 group M, lending support to the decisive role of GUD-enhanced transmission in the emergence of this pandemic virus. We believe this new information will be useful to other researchers investigating the origin of HIV and to researchers interested in the ways retroviruses are able to adapt to new host species. Understanding why the origin of HIV shows unique timing patterns in comparison with other retroviruses is important to prevent future epidemics and pandemics.

Our research about the origin of HIV might have implications for the origin of other pandemic pathogens, including SARS-CoV-2. Human exposure to SIV and non-human coronaviruses does not seem a limiting factor in either case: exposure of many thousands or even millions of humans to bushmeat has been going on for millennia. HIV could not have emerged without profound societal changes, including urbanization and social disruption initiated by colonialism, which allowed CSW and GUD to flourish. Likewise, what made the current COVID-19 pandemic possible was, in part, societal changes, including the intensity of travel and probably urbanization, which multiplied the contacts of the susceptible population.

In addition, our research allowed us to define a template for *adaptation* of zoonotic viruses to the human host: co-factors that help a certain kind of virus to spread between humans, if unusually concentrated in time and space, may ignite the serial transmission of a non-adapted zoonotic virus of a similar kind, and this process enables adaptation, as demonstrated in experiments, such as with SIV and H5N1 [24,32]. We propose that GUDs and other STDs, associated with CSW, concentrated in time and space, ignited such an adaptive process, giving rise to HIV-1. Similarly, SARS-CoV-2 may have adapted to humans by selective processes operating after zoonotic transfer [93]. The process we propose for HIV may apply to the adaptation of SARS-CoV-2, other coronaviruses, influenza viruses, and other respiratory tract viruses. The risk of epidemics or pandemics may be higher if, wherever bushmeat or poultry is handled, people are overcrowded, have respiratory tract bacterial or fungal infections, inflammatory states, or are shouting aloud or behaving in a way that would promote viral transmission. Such conditions might allow an initially poorly transmitted zoonotic strain to adapt to humans by serial transmission, and the high social mobility of modern times will then give it a chance to become pandemic.

## Data Availability

The data presented in this study are available in Supplementary Dataset S1.

## Supplementary Materials

The following are available online at https://www.mdpi.com/article/10.3390/v13091701/s1, Text S1: Supplementary Text S1 (this file contains Tables S3–S5): Main Supplementary Information, Table S1: List of nosological tables and sources, Table S2: List of demographic information and sources, Figure S1: Proportion of primo-secondary syphilis over time, Dataset S1: Demographic data and counts of treated STD cases, Script S1: R script using smooth function to estimate proportion of primo-secondary syphilis.
