# Supplementary material for: Newly Discovered Archival Data Show Coincidence of a Peak of Sexually Transmitted Diseases with the Early Epicenter of Pandemic HIV-1
Source: Viruses. 2021 Aug 27;13(9):1701. doi: 10.3390/v13091701 (PMC8472979; doi:10.3390/v13091701)
Supplement: Supplementary file 1 [file viruses-13-01701-s001.zip › Suppl_Text_S1.pdf]

Supplementary Information

Newly discovered archival data show coincidence of a peak of sexually transmitted diseases with the early epicenter of pandemic HIV-1

João Dinis Sousa [1,2], Philip J. Havik [2], Viktor Müller [3] and Anne-Mieke Vandamme [1,2,4]

[1] Clinical and Epidemiological Virology, Rega Institute for Medical Research, Department of Microbiology, Immunology and Transplantation, KU Leuven, B-3000 Leuven, Belgium; annemie.vandamme@kuleuven.be
[2] Global Health and Tropical Medicine, Instituto de Higiene e Medicina Tropical, Universidade Nova de Lisboa, 1349-008 Lisbon, Portugal; philip.havik@ihmt.unl.pt
[3] Institute of Biology, Eötvös Loránd University, 1117 Budapest, Hungary; mueller.viktor@ttk.elte.hu
[4] Institute for the Future, KU Leuven, B-3000 Leuven, Belgium

Supplementary Information

1. Sources used for the nosological tables and demographic data

To obtain disease case counts and demographic data on Leopoldville, we consulted the African Archives of the Belgian Ministry of Foreign Affairs, which absorbed the Archives of the former Ministry of Colonies (Afrika-Archief, Federale Overheidsdienst – Buitenlandse Zaken, Buitenlandse Handel en Ontwikkelingssamenwerking, FO-BZBHO) and additional sources. Most of these sources are periodical annual reports [42–52]. For a full list of all sources, see Supplementary Table S1 (sources for the nosological tables) and Supplementary Table S2 (sources for the demographic data), provided in Excel format. In all this Supplementary Information, as well as in Supplementary Tables, reference numbers correspond to the reference list of the main article.

1.1. Printed periodicals

Many of the printed periodicals were obtained by means of photocopies, electronic copies, or notes taken while consulting them the Afrika-Archief, or in the Afrika-Archief library (distinct from the Archive itself). Of these reports, some series have been published for a wider readership. They are listed in Supplementary Table S3. In both Supplementary Tables S1 and S2, in the columns called "Boxes or sources" we list the sources consulted for each year, including, if appropriate, the codes of the published periodicals, as listed in the first column of Table S3.

Supplementary Table S3. The published periodicals referred to in Supplementary Tables S1 and S2. The title presented here is the most representative one, there were slight variations between the years.

Code in Tables S1 and S2	Representative title	Reference numbers
ASBCB	Annuaire Statistique de la Belgique et du Congo Belge	[50]
BOCB	Bulletin Officiel du Congo Belge	[51]
CRC	Croix Rouge du Congo. Rapport Annuel	[42]
RACB	Rapport sur l'Administration de la Colonie du Congo Belge	[46]
RHPCB	Rapport sur l'Higiène Publique au Congo Belge	[47,48]
RADGSM	Rapport Annuel de la Direction Générale des Service Médicaux du Congo Belge	[49]

Table S3 only contains the periodicals that were published. Additional periodical reports exist (for example, on the health conditions in a province, in the District Urbain the Leopoldville (DUL), or in a hospital) that apparently were not published (probably they circulated only among administrative personnel in both the Congo and Belgium). We consulted these additional unpublished reports in the Afrika-Archief.

1.2. Archival, non-published, sources

The archival sources are all inside boxes that can be ordered for consultation in the Afrika-Archief. In the columns "Boxes or sources" we identify archival, non-published sources, starting with an underscore. The typical content of these boxes is made up of reports, some of them periodical, often many in each box. But they also contain non-periodical reports written by a physician, for example, and other documents, and correspondence. Boxes are organized in inventories. Table S4 lists all the inventories and the corresponding link to the boxes we consulted with them.

Supplementary Table S4. The Afrika-Archief inventories for which we consulted boxes referred in Supplementary Tables S1 and S2.

Inventory	Boxes that we consulted and are referred in Tables S1 and S2
A39	_80, _81, _82, _85, _87, _88, _89, _112, _135, _136, _252, _253, _254, _D379
A39RA	_RAMED16, _RAMED17, _RAMED18, _RAMED46, _RAIMO119, _RAAIMO119an, _RAAIMO120, _RAAIMO120an, _RAAIMO228
A34	_3545
A34bis	_1985
A11	_4388-831, _4389-841, _4390-179, _4393-202, _4393-204, _837
GG	_GG916, _GG5398, _GG5832, _GG16807, _GG16819, _GG16823, _GG16829, _GG16830, _GG16862, _GG16864
RACBGG	_RACBGG239, _RACBGG513, _RACBGG514, _RACBGG553
RACCB	_RACCB6, _RACCB7, _RACCB9, _RACCB9bis, _RACCB10, _RACCB11, _RACCB12, _RACCB13, _RACCB14, _RACCB15, _RACCB16, _RACCB17, _RACCB961, _RACCB965, _RACCB972, _RACCB1089, _RACCB1046Leo

1.3. Additional published sources

The additional published sources figuring in Supplementary Tables S1 and S2 are articles and books. We indicate the first author and the year of publication and list them in the reference list of the main article.

1.4. Notes to Supplementary Table S1

This table aims to list the sources we consulted to obtain disease counts from nosological tables or equivalent information. It is divided in three main sets of columns, for colony-level, province-level, and DUL (Leopoldville city)-level data, respectively. As we write in Methods, although we were mainly interested in DUL-level data, the latter is often present in province-level or even colony-level reports.

The columns "Boxes or sources" indicate the boxes of the Archives in which the information exists. The "Type" column indicates the broad type of report. Often a certain report is present in more than one box, or even is a published report that has a copy inside a box. For example, in the line of the year 1916 we identify a "*Rapport d'Hygiène*" ("rhyg") that belongs to a published periodical series, "RHPCB" (see [47,48] and Table S3), a copy of which 1916 volume is also contained in the box "_81". This is a colony-level report that could potentially have 7 nosological tables, for colony-level, province-level, and DUL-level disease counts, separately for Africans and Europeans (which gives 6), and a 7th for *Croix Rouge du Congo* [42] disease counts. In fact, it has only 3 nosological tables, all for Europeans, colony-level, province-level, and Leopoldville city-level. The corresponding tables for Africans are absent and hence these cells are marked with "x". The "HN" code means that the table is for Hospitalized or Not ("*Hospitalisés ou Non*") patients. The "HO" code means a nosological table is present for Hospitalized Only ("*Uniquement Hospitalisés*") patients. Since we are studying STD incidence, we are most interested in HN nosological tables. We still list the "HO" and "x" occurrences in Table S1 to show that we actively searched the archives for that year and could not find an HN table.

As Supplementary Table S1 shows, the nosological tables in question pertained to three different 'health sectors' (or 'health systems') operating in the city: Government, or Public Sector, *Croix Rouge du Congo* (CRC), and Industrial Hygiene (IH), i.e. healthcare provided by private enterprises. We make further reference to these data in section 2.

Some of the references in the table are of published periodical reports; in that case, they are listed in the reference list of the main article.

1.5. Notes to Supplementary Table S2

The relevant demographics only refer to the DUL (or Leopoldville city), and the total annual population count, which is also partitioned in men, women and children. The sources containing this information are listed in Supplementary Table S2, in the columns "boxes or sources". The codes in these columns follow the same rules as in Supplementary Table S1. Some of the sources are books and articles, listed in the reference list of this Supplement.

The "Total" and "Partition" columns contain "RC" if the data is provided by an official census or statement, "RE" if it is an estimate made by the author of the listed source, and "x" if no data is available.

For Africans population counts, only DUL-level data is relevant. For European population counts, in addition to DUL-level data, we also list data on the district of Moyen Congo (period

1906–22) because it is used by us to estimate missing DUL-level data for some years. For Europeans, we also list province-level data (Congo-Kasai province until 1932 and Leopoldville province from 1933 onwards) because we use these data to build Figure 2 of the main article with incidence of treated cases of STDs in Europeans of the province.

Some of the references in the table are of published periodical reports, articles and books; in such cases, they are listed in the reference list of the main article.

2. Disease counts in Africans

2.1. The different health services and relation with Supplementary Table S1

In Leopoldville, only three health care sectors operated during the period under consideration: public, government based, the *Croix Rouge du Congo à Léopoldville* (CRC; considered a philanthropic organization) and services run by private enterprises. Each health report published by the colonial administration includes a principal nosological table with the cases treated by the Public Sector (PS) led services only. Starting in 1923, most *Hygiène Publique* reports also have a section on *Hygiène Industrielle*, or Industrial Hygiene (IH), providing a nosological table for those treated by private enterprises. The cases treated in CRC (between 1929, when this clinic opened, and 1954, when it was absorbed by the Public Sector) were reported in CRC reports and often also in separate sections of the other reports. This allowed for the creation of a non-overlapping dataset: none of the cases of a disease reported for the DUL was referred in multiple sections or tables; if reported in the main table, it was a PS case; if in the *Croix Rouge* reports, a CRC case, and certainly not overlapping with PS cases, since the *Croix Rouge* was a philanthropic organisation; if reported in the IH table, it was neither PS nor CRC.

Although there were two hospitals, twelve health dispensaries/clinics, and several other health facilities treating Africans in the city by 1937, they all belonged to one of the three categories above [52]. Therefore, for each disease, the sum PS + CRC + IH, reliably provides the number of treated and recorded cases in DUL.

CRC tables start in 1929 (because the CRC clinic opened in that year) and IH tables start in 1923. We only consider that we have complete data for Africans for a given year and a given STD if we have Hospitalized or Not (HN) counts of that STD for all services active in that year. For example, let us concentrate on the year 1931. Since the CRC and IH systems were already put in place by 1931, we require the existence of all three DUL-level HN tables (of Public Sector, Croix Rouge, and Industrial Hygiene) to ascertain that the said year has valid data. And, as can be seen in Supplementary Table S1, this is the case for 1931: the DUL-level PS table is present in the province-level report stored in box _88, the CRC table exists in two different boxes (_GG5398 and _RAMED46) and also as a published printed report, and the IH table exists in boxes _88 and _RAMED46. Thus, the year 1931 has complete data.

For a second example, focusing in the year 1919, we can see in Supplementary Table S1 that the DUL-level Public Sector nosological HN table can be found in different sources: in colony-level reports stored in several boxes and in a province-level report stored in box _RACBGG513. The CRC and IH tables do not exist because these services had not been created yet in 1919. Thus, our 1919 data is also complete.

It is important to add the caveat that colonial health statistics cannot be taken at face value. Several biases existed. First, at least until 1929, many Africans with STDs were not treated, this being more pronounced for women. We found a marked male-biased sex ratio in hospital

consultations, exceeding population sex ratio. The diagnoses of STDs were mostly clinical, leading to confounding between syphilis and chancroid and between tertiary syphilis and tertiary yaws. "Blennorrhagia" was a commonly used term, encompassing both gonorrhea and chlamydia, since the distinction between the two was ignored in early 20th century. Serological tests existed only for syphilis until the 1930s, and they did not distinguish syphilis from yaws. We explain these and other biases elsewhere [40]. The confusion between syphilis and yaws was only likely in tertiary or purely serological diagnoses and not in primo-secondary syphilis. The absence of distinction between gonorrhea and chlamydia in the reports does not invalidate our data series, because we also do not attempt to estimate gonorrhea and chlamydia separately and gather the counts of "blennorrhagia". In addition, Africans adapted their health seeking behavior when STD treatments improved. For a full discussion, see [40].

2.2. Estimates of missing data

For the years before 1919, we can see that there are no DUL-level HN tables for Africans (Supplementary Table S1). After many searches in the Afrika-Archief spanning 12 years we could not find them, probably because they do not exist. Before 1919, colonial officials were reporting only Hospitalized Only (HO) tables for Africans. Therefore, in both our 2010 article [6] and in the present article, we start our main series of incidence of treated cases in Africans in 1919.

Our treatment of missing data here is similar to the methods we used in our 2010 and 2021 articles [6,40]. To start with, for each year, a set of services is active: 1) for 1919–22, only Public Sector; 2) for 1923–28 and for 1955–58, Public Sector and Industrial Hygiene; 3) for 1929–54, Public Sector, Industrial Hygiene and Croix Rouge.

If, for a given STD and for a given year, relative to the health services active for that particular year, data was missing *for more than one* service, we rejected that year. For example, for syphilis, and for 1942, we have data for CRC but not for PS or IH; therefore, we rejected that year for syphilis.

For a given STD and a given year, if data was lacking *for only one* active service, we made an estimate of the data for that service. We list the estimates made below.

3. Disease counts in Europeans

3.1. The main series of data available

Europeans were mostly treated by the Public Sector. Some were also treated by private firms but the corresponding disease counts are not available. Therefore, we collected Public Sector HN nosological tables at DUL-level for them, from the same sources that present nosological tables for Africans (colony-level, province-level, and DUL-level health and other reports). The resulting HN tables are also listed in Supplementary Table S1.

We were able to find such HN tables for most years of the period 1915–35 (with few years missing). For the period 1936–58 the reports cease to provide HN tables at the DUL-level, although they do so for province-level HN tables.

3.2. Estimates of missing data

For the years 1906–1914 we obtained many reports containing tables we label as "HM" – Only Hospitalized Only case counts presented in table but there are additional comments with quantitative content about non-hospitalized cases. After careful analysis, we decided to make

estimates only for syphilis, and only for three of these years: 1910, 1911 and 1914, based on the following data.

In 1910, Dr Bourgoignie saw 18 syphilitic primary chancres in European agents in one semester [79]. This provides our lower estimate for the entire year. We assume that the other semester would have the same number. One contemporaneous source says that about 1/3 of European colonial military treated for syphilis were seen in the primary stage [85]. If we assume also that half of Europeans contracting syphilis would present themselves for treatment in the primary stage, the other half presenting secondary stage syphilis (which is reasonable because the Europeans recruited for the colony at that time are described as being careless about STDs [79,80]), there would have been 72 treated cases in 1910. This is our upper estimate.

In 1911, 46 syphilis cases in Europeans were treated in the hospital (but not all hospitalized) and additional cases were treated at home [80]. Based on this, our lower estimate equals the reported count, 46, and our upper estimate is 70.

In 1914, a report says that 105 injections of *NeoSalvarsan* or *Salvarsan* were administered to European syphilis patients (both hospitalized and non-hospitalized), in that year, without specifying the number of patients [83]. In the Dr Mouchet cohort, each patient received 1.65 *Salvarsan* injections on average [84]. In the Dr Noc cohort of colonial troops (in French Martinique), in 1911, the average was 1.97 per patient [87]. We assume that the maximum used at that time, in colonial settings, was around ~3 per patient and the minimum was the 1.65 from Dr Mouchet, and so the 105 injections above cited correspond to a lower and upper estimates of treated syphilis cases of 35 and 64, respectively.

4. Demographic data on Africans

Supplementary Table S2 contains references to sources of demographic data, both archival and others [16–22]. It includes uninterrupted series of demographic data on Africans at DUL-level for the period 1919–58. This includes both total counts and partition in men, women and children. For all these years, the data is provided by either censuses or official statements (code "RC").

Importantly, the breakdown of data in men, women and children is necessary, because we compute incidence of treated cases in adults (for syphilis and discharge syndrome) and in adult males (for chancroid and venereal bubo); see Methods.

For all years, a breakdown of the population count between the two main parts of the city (Leopoldville-East and -West) is available. For some years, the population of the quarter of the African military force and their families (*Camp Militaire*, a minor quarter compared to Leopoldville-East and -West) is also available. When that part was missing, we estimated it, mostly by linear interpolation. We then summed up all three parts to obtain total counts for each year.

We have less complete data for the pre-1919 years but this turns out to be irrelevant because we do not have pre-1919 complete STD data for Africans, and so we start our incidence series in 1919 ([6,40]; see also Figure 1 of the main article).

5. Demographic data on Europeans

As Supplementary Table S2 shows we have demographic data on Europeans in Leopoldville/DUL for many years throughout the period 1906–58, but data is missing for a number of years. However, we only have STD data at DUL-level for the period 1910–35 (this can

be seen in Supplementary Table S2 and in our few additional STD count estimates described in section 3.2). Therefore, we only needed to estimate missing demographic data for the period 1910–35. The missing years were 1911–18 and 1921–22.

We calculated the average of the ratio Leopoldville city/Moyen Congo district for the years 1906–10, since these data are available (Supplementary Table S2) as being 0.729. The Moyen Congo was a small district that encompassed Leopoldville; from 1923 onwards, Leopoldville itself was transformed into a district (the DUL). We then applied that ratio to the Moyen Congo population and estimated the Leopoldville population for 1911.

The said ratio for 1919 was 0.836. We used linear interpolation between the 1911 and the 1919 ratios to estimate the Leopoldville/Moyen Congo ratios for the period 1912–18. For some years of that period the Moyen Congo population count was missing. We estimated it by linear interpolation. The use of such interpolation is justified because typically the population does not change much during periods of one to three years. Applying the above cited ratios, we estimated the European population of Leopoldville for the whole period 1912–18.

We then calculated the average of the ratio Leopoldville city/Moyen Congo for the years 1919–20. We applied that ratio to the Moyen Congo counts of 1921–22 and estimated the European population of Leopoldville for 1921–22.

Then we proceeded to estimate missing data for the proportion of adults and men. The proportion of adults is very stable at 96–97% for the years 1910, 1919, 1925, and 1926. Thus we used it for all estimates within this period. We subsequently applied the ratio 0.965 to the Leopoldville population count and estimated the adult count for the years within the period 1911–24 for which the data was missing (i.e., years for which no data on breaking down of population is available).

We needed to estimate the number of men for the years 1920–24 (we did not need it before 1920 because number of men is only needed to calculate incidences of chancroid and venereal bubo, and data on these diseases is not available before 1920). We calculated the proportion of men in 1919 and 1925, linearly interpolated this proportion for 1920–24 and estimated the number of men for these years.

For the province as a whole, we used the same methods of interpolation of counts, proportion of adults, and proportion of men.

6. Proportion of treated syphilis that was primary and secondary

For many years, the reports break down the treated syphilis cases by stage of syphilis. In that case, we calculated the proportion of primo-secondary syphilis (PSS), P_{pss} as:

$$P_{pss} = (N_{ps} + N_{ss}) / N_{as}$$

Being N_{ps}, N_{ss} and N_{as} the numbers of cases of primary syphilis, secondary syphilis, and syphilis at any stage, respectively. This measure is important because syphilis causes genital ulcers only in these two stages. We were able to obtain data on partition by syphilis stages for the years 1912, 1926–34, 1945, 1949–51, 1954, 1956–58. For the years 1926–29, only data at the level of the province was available and, in these years, we assumed the DUL had a partition with 10% lower PSS proportion than the province, broadly supported by a contemporaneous article [71]. For the years 1930–34, the reports mentioned “secondary and tertiary” together in a single category; in such cases we assumed secondary was ½ of the number described as “secondary and tertiary” because the proportions of secondary and tertiary were approximately the same in neighboring years for

which we have their proportions discriminated. For the years for which no partition data was found, we imputed it using a smooth function constructed through local polynomial regression fitting over the available data points, as implemented in the loess() function of the R language [64]. The R script is provided as Supplementary Script S1. Figure S1 shows the temporal evolution of PSS proportion, both raw data and our smooth function.

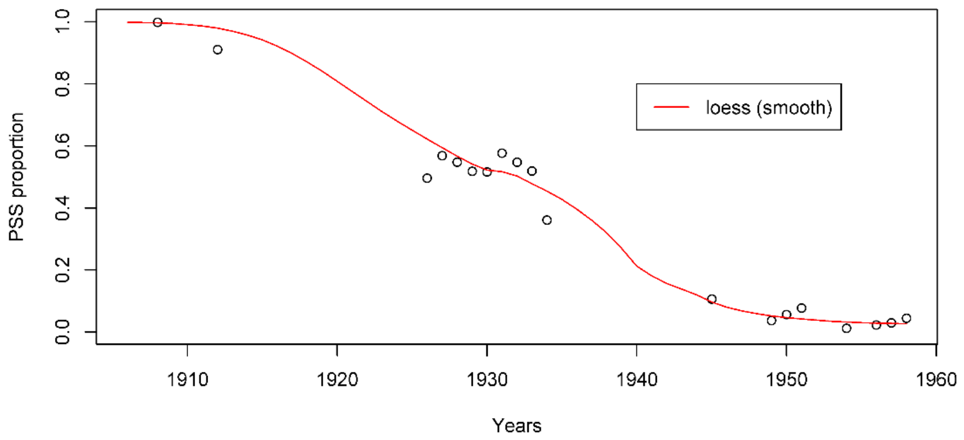

Figure S1. The evolution of the proportion of syphilis cases that were at primary or secondary stage (PSS) over time. Points show existing data on this proportion after the corrections described in the main text. The continuous line was obtained through a smooth function described in the main text.

As Figure S1 shows, proportion of PSS declined monotonically throughout the period 1910–60, from >90% in the 1910s to <10% in the 1950s. As syphilis epidemics progressed, an increasing fraction of infected patients were seen with tertiary symptoms and also, as serological screening expanded, an increasing fraction of diagnoses were purely serologic. The same pattern was seen in other colonies for which we also researched STD data. The pattern is also predicted by epidemiological simulations of syphilis [69].

7. Estimate of male circumcision rate

In our 2010 paper we collected information on male circumcision practices per ethnic group from ethnographic sources and we collected information on the ethnic partition of the African population of several cities at several time points [6]. For Leopoldville, 1919, these methods allowed us to calculate lower and upper estimates of the circumcision rate (C_a) of 73.5% and 82.5%. The methods are explained in detail in that article [6].

In the present work we recalculate male circumcision rate for the whole population including Europeans. In early 20th century, male circumcision in non-Jewish Europeans outside the United Kingdom was nearly absent [65,66]. Very few Europeans present in Leopoldville were British or Jewish. Therefore, we estimate 2% circumcision rate (C_e) in them. There were N_a = 11,021 African men and N_e = 495 European men in the city, in 1919. Therefore, the total circumcision rate is:

$$C = (N_a * C_a + N_e * C_e) / (N_a + N_e)$$

This gives lower and upper estimates of 70.4% and 79.0%, respectively.

8. The dataset supporting Figures 1 and 2

The data supporting Figures 1 and 2 is shown in Supplementary Dataset S1. It contains, for both Africans and Europeans, demographic data, data on counts of treated STDs, and incidence of treated cases.

9. Complementary evidence showing high levels of STDs in the period 1910–19

The following Supplementary Table S5 lists statements by colonial physicians demonstrating high levels of STDs in both Europeans and Africans, period 1910–20.

Suppementary Table S5. The statements in various reports supporting incidence rates of STDs in the period 1910–19, in Leopoldville, in both Africans and Europeans.

Li-ne	Year	Author	Ref.	Statement	Notes
1	1910	Dr Bourgoignie	79	He saw 18 syphilitic primary chancres in European agents in one semester	290 adult European residents in Leopoldville; this implies a ~19% annual incidence (see section 7.1)
2	1910	Dr Bourgoignie	79	50% of Europeans "have" STDs	Probably means in the last semester or in the last year
3	1911		80	The hospital treated 46 syphilis cases in Europeans and additional cases were treated at home	359 adult European residents in Leopoldville
4	1911		80	STDs are most important cause of morbidity in Europeans	They mean that STDs together surpass the second most important category (malaria)
5	1912	Dr Houssiau	81	In 80–90 European State agents he saw 40–45 cases of venereal diseases during the year	Consistent with line 2
6	1911–1912		76–78	Monthly screening of African workers finds 712 cases of urethral discharge ("blennorrhagie") in 7 months	Average of 1124 African workers; annual incidence ~109%
7	1911–1912		76–78	Monthly screening of African workers finds 46 cases of syphilis in 7 months	Average of 1124 African workers; annual incidence ~7.0%
8	1913	Dr Mouchet	82	Almost all African workers had discharge in a period of one year	Consistent with line 6
9	1926	Dr Kadaner	44	STDs incidence rates in ~1920 were much higher than in 1926	

9.1. Notes on Supplementary Table S5

In 1910, Dr Bourgoignie saw 18 syphilitic primary chancres among European personnel in one semester, at a time when only 290 European adults were living in Leopoldville [79]. Some of these cases could be among the numerous non-resident personnel (floating population), since the city was constantly receiving European visitors stationed in other parts of the colony. The number is nevertheless staggering, because by that time, most syphilis cases were not detected in the primary stage [84,815. Assuming about 1/3 of cases were seen in their primary stage, as in a contemporaneous statistic of European colonial military personnel [85], that would mean ~54

syphilis cases in a semester, or ~108 in a year. Even with the unlikely assumption of a floating population as large as the resident one, the total population covered would be ~580, and the treated syphilis annual incidence would be ~19%. Such high syphilis incidence rates were similar to those seen in colonial troops in close contact with CSWs [85,86].

And surveys of Leopoldville's African public sector workers made in 1911–12 showed incidence rates of urethral discharge (‘*blennorrhagie*’) far higher than in any of the nosological tables from 1919 onwards: 712 entries of ‘*blennorrhagie*’ and 46 of syphilis detected in 1,124 men during a seven month period [76–78], suggesting annual incidence rates of treated discharge of 109% and of treated syphilis of 7.0% (although they could be lower than that because these reports were monthly and a single case in an individual could give more than one entry in consecutive months). Of note here is that these data are not comparable to those depicted in Figures 1 and 2 of the main text, because they refer to male workers subject to very intensive screening (about once per month).

There are several statements in archival records of STDs being diagnosed in 50% of Europeans, either in a one-year period or for an undefined period lasting several months (Table S5).

10. References

The references cited in this Supplementary Information are listed in the reference list of the main article.
