# Supplementary figures and images for: Newly Discovered Archival Data Show Coincidence of a Peak of Sexually Transmitted Diseases with the Early Epicenter of Pandemic HIV-1
Source: Viruses. 2021 Aug 27;13(9):1701. doi: 10.3390/v13091701 (PMC8472979; doi:10.3390/v13091701)

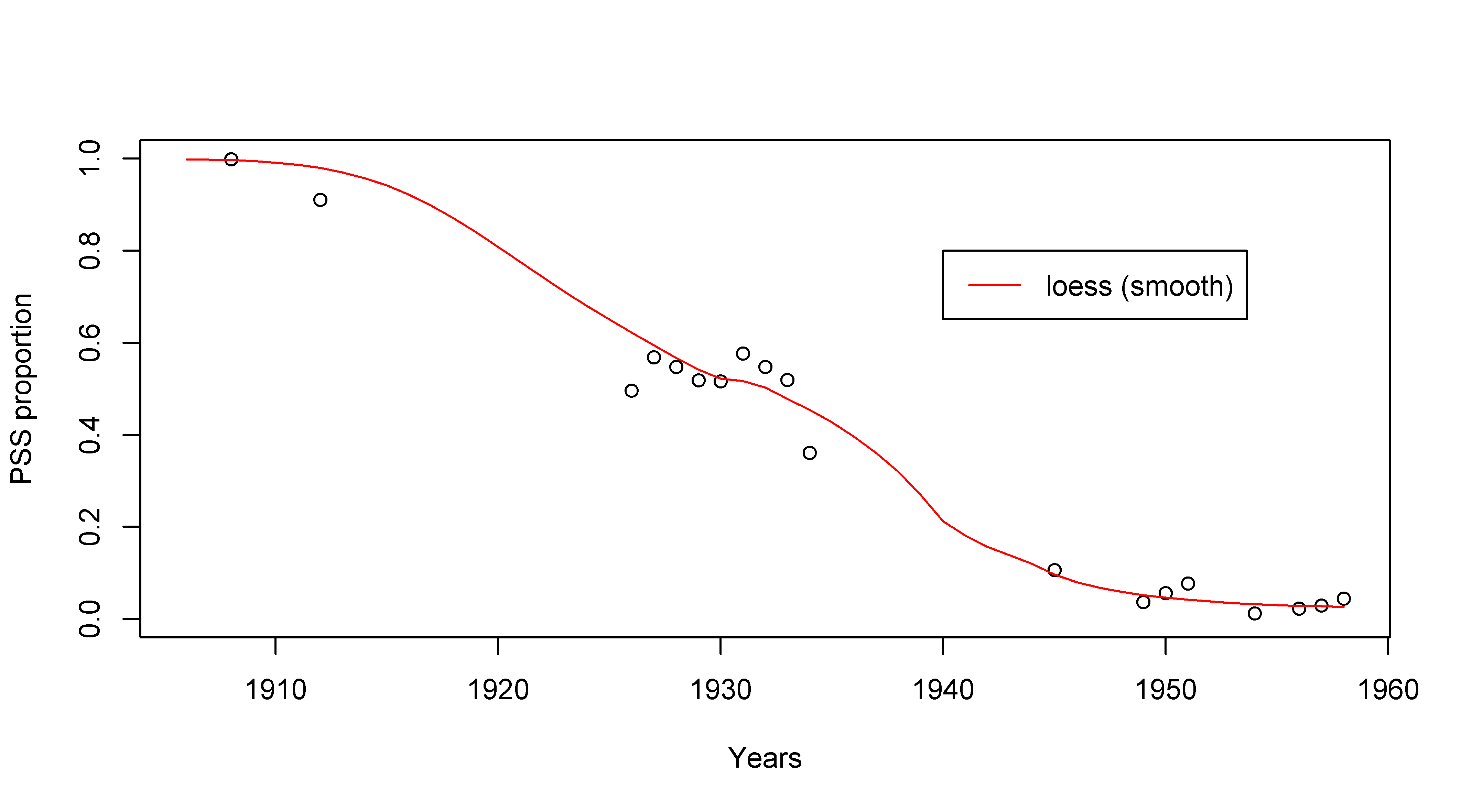

Supplement: Supplementary file 1 [file viruses-13-01701-s001.zip › Suppl_Fig_S1.tif]
